# Bone resorption and remodeling in murine collagenase-induced osteoarthritis after administration of glucosamine

**DOI:** 10.1186/ar3283

**Published:** 2011-03-16

**Authors:** Nina Ivanovska, Petya Dimitrova

**Affiliations:** 1 Department of Immunology, Institute of Microbiology, 26 Georgi Bonchev str., Sofia, 1113, Bulgaria

## Abstract

**Introduction:**

Glucosamine is an amino-monosaccharide and precursor of glycosaminoglycans, major components of joint cartilage. Glucosamine has been clinically introduced for the treatment of osteoarthritis but the data about its protective role in disease are insufficient. The goal of this study was to investigate the effect of long term administration of glucosamine on bone resorption and remodeling.

**Methods:**

The effect of glucosamine on bone resorption and remodeling was studied in a model of collagenase-induced osteoarthritis (CIOA). The levels of macrophage-inflammatory protein (MIP)-1α, protein regulated upon activation, normal T-cell expressed, and secreted (RANTES), soluble receptor activator of nuclear factor kappa-B ligand (RANKL), tumor necrosis factor (TNF)-α, and interleukin (IL)-6, 4 and 10 in synovial fluid were measured by enzyme-linked immunosorbent assay (ELISA). Cell populations in synovial extracts and the expression of RANKL, of receptors for TNF-α (TNF-αR) and interferon γ (IFN-γR) on clusters of differentiation (CD) three positive T cells were analyzed by flow cytometry. Transforming growth factor (TGF)-β3, bone morphogenetic protein (BMP)-2, phosphorylated protein mothers against decapentaplegic homolog 2 (pSMAD-2), RANKL and Dickkopf-1 protein (DKK-1) positive staining in CIOA joints were determined by immunohistochemistry.

**Results:**

The administration of glucosamine hydrochloride in CIOA mice inhibited loss of glycosaminoglycans (GAGs) and proteoglycans (PGs) in cartilage, bone erosion and osteophyte formation. It decreased the levels of soluble RANKL and IL-6 and induced IL-10 increase in the CIOA joint fluids. Glucosamine limited the number of CD11b positive Ly6G neutrophils and RANKL positive CD3 T cells in the joint extracts. It suppressed bone resorption via down-regulation of RANKL expression and affected bone remodeling in CIOA by decreasing BMP-2, TGF-β3 and pSMAD-2 expression and up-regulating DKK-1 joint levels.

**Conclusions:**

Our data suggest that glucosamine hydrochloride inhibits bone resorption through down-regulation of RANKL expression in the joints, via reduction of the number of RANKL positive CD3 T cells and the level of sRANKL in the joints extracts. These effects of glucosamine appear to be critical for the progression of CIOA and result in limited bone remodeling of the joints.

## Introduction

Glucosamine is one of the most abundant amino-monosaccharides immediately phosphorylated and included in a hexosamine biosynthesis pathway. The end-product of this pathway is UDP-N-acetylglucosamine, which is important for the synthesis of glycoaminoglycans and glycolipids. Exogenous glucosamine is bound with high affinity to glucose transporter GLUT-2 [1] and can induce insulin resistance in adipocytes [2] and skeletal muscle cells [3]. In joint and cartilage, glucosamine can regulate the metabolism of glycosaminoglycans favoring catabolic processes. Glucosamine expresses a number of *in vitro *effects on chondrocytes, including stimulation of proteoglycan synthesis, inhibition of proteoglycan and collagen degradation, suppression of IL-1 induced activation and decrease of NF-κB activity [4-8]. Glucosamine has anti-inflammatory action suppressing inducible nitric oxide synthase (iNOS) expression [9,10], neutrophil functions [11], activation of T-lymphoblasts and dendritic cells [12].

Glucosamine has been used for the treatment of osteoarthritis (OA). It is administered in different pharmacological forms, including sulfate, N-acetyl-glucosamine, or chlorohydrate salt [13]. Oral application of glucosamine is more frequent, but experimental data about the effect of its intravenous injection have also been performed [14]. Glucosamine is absorbed from the gastrointestinal tract [15]. Depending on the pharmacological form used, its half-life in serum is from 28 h to 58 h [14]. Müller-Fassbender *et al*. have established that glucosamine sulfate is as effective as ibuprofen in patients with knee OA [16]. Long-term oral treatment with this pharmacological form delayed the progression and improved the symptoms of knee osteoarthritis acting as a disease modifying agent [17]. In different trials, it has been reported to exert improvement in OA [18], have a moderate effect [19] or show no difference with a placebo [20,21]. This variation in the results determines a need of more systemic investigations on the mechanisms of glucosamine action.

OA develops as a result of an imbalance between bone resorption and bone remodeling. Therefore, we have conducted this study to evaluate the effect of glucosamine on these processes mainly in the joint. Studies on its systemic effects were not in the focus of our experiments. Glucosamine was administered in an animal model of OA. We determined the levels of pro- and anti-inflammatory mediators and the phenotype of cells in the synovial extracts as well as the expression of resorption and remodeling markers in the joints.

## Materials and methods

### Mice

Outbred ICR (CD-2) male mice, 10- to 12-weeks-old, weight 20 to 22 g, were purchased from the Charles River Laboratories (Wilmington, MA, USA). Mice were maintained on a 12:12 h light:dark cycle and fed standard diet and tap water *ad libitum*. All experiments were conducted in accordance with the Bulgarian National Guidelines for the Care and Use of Laboratory Animals (Decree No. 14/19.07.2000) and were approved by the Animal Care Committee at the Institute of Microbiology, Sofia.

### Collagenase-induced osteoarthritis (CIOA) and treatment

For induction of OA, ICR male mice were injected at right and left knee intra-articular (i.a.) space with 1 U/10 μl or with 2 U/10 μl of collagenase from *Clostridium histolyticum *(Sigma-Aldrich, Diesenhofen, Germany) at days 0 and 2. The incidence of OA was approximately 90%. A study to compare the development of CIOA in male and female animals was not conducted. Control group of animals received i.a. injection of 10 μl endotoxin-free phosphate-buffered saline (PBS; Lonza, Verviers, Belgium).

D(+)-glucosamine hydrochloride (Glu) and D-glucosamine 2-sulfate sodium salt (GS) purchased from Sigma-Aldrich (Munich, Germany) were dissolved in sterile PBS and were administered orally by gavage at a dose of 20 mg/kg/daily. Two groups were treated with Glu (CIOA + Glu1) or GS (CIOA + GS) for 20 days, starting from Day 7 after the second collagenase injection and a group of mice with arthritis were fed with PBS (CIOA). Control groups of mice were i.a. injected with PBS and were untreated (healthy) or treated with glucosamine hydrochloride (healthy + Glu). One group of mice was treated with Glu for 20 days starting with the second collagenase injection (CIOA + Glu2). In another experimental setting CIOA was induced after injection of a higher dose of collagenase (2 U/mouse at Day 0 and at Day 2). The oral administration of glucosamine hydrochloride started 7 days after the second collagenase injection and lasted 20 days.

### Synovial extracts

Patellae with surrounding soft tissue (tendon and synovium) were excised, and incubated in 200 μl of serum-free RPMI 1640 medium (Biowhittaker™, Lonza, Verviers, Belgium) for 2 h at 37°C as described by van de Loo *et al*. [22]. The washouts were collected separately for each animal and centrifuged at 1,200 × *g *for 10 minutes. Supernatants were stored at -70°C and used for cytokine assays. Synovial cells were counted and used for flow cytometry analyses.

### Determination of chemokines and interleukins in synovial extracts

The amounts of MIP-1α, RANTES, soluble RANKL, TNF-α, IL-6, IL-4 and IL-10 in synovial extracts were determined by ELISA using mouse ELISA kits. The samples were assayed in triplicates. The concentration of chemokines and cytokines was calculated from a standard curve of the corresponding recombinant mouse protein, using Gen5 Data Analysis Software (BioTek Instruments, Bad Frienarichsall, Germany) and was presented in pg/ml supernatant. ELISA kits were with detection limits 8 pg/ml for MIP-1α, 16 pg/ml for RANTES, 62 pg/ml for RANKL, 32 pg/ml for TNF-α, 20 pg/ml for IL-6, 20 pg/ml for IL-10 (all from PeproTech EC, London, UK) and 10 pg/ml for IL-4 (BD Pharmingen, Erembodegem, Belgium).

### Isolation of peripheral blood mononuclear cells (PBMCs)

Blood was collected by retro-orbital puncture in tubes containing 5 U/ml of heparin. Blood was mixed with an equal volume of PBS (pH 7.4). After gradient centrifugation on Histopaque 1083™(Sigma-Aldrich) at 1,400 × *g *for 40 minutes at room temperature, peripheral blood mononuclear cells (PBMCs) were carefully collected, washed with PBS, counted and used for flow cytometry analyses.

### Flow cytometry

Synovial extract cells and peripheral mononuclear cells were resuspended at 2 × 10^5^/ml in PBS containing 2% fetal calf serum (FCS). The cells were incubated for 15 minutes at 4°C with appropriately diluted antibodies from BD Pharmingen (Erembodegem, Belgium) against mouse Ly-6G (FITC-conjugated; clone RB6-8C5), CD11b (PE-conjugated; clone M1/70), CD69 (APC-conjugated, clone H1.2F3) and CD3 (FITC or PE conjugated, clone 145-2C11) washed and used in flow cytometry.

For determination of TNF-αR, IFN-γR and RANKL expression, synovial cells and PBMCs were incubated for 30 minutes at 4°C with biotinylated antibodies against mouse TNF-αR1 (2 μg/ml; clone H-271; Santa-Cruz Biotechnology Inc., Heidelberg, Germany), IFN-γRα (5 μg/ml; clone M-20; Santa-Cruz Biotechnology) and RANKL (1 μg/ml, PeproTech EC) or with isotype controls (Sigma-Aldrich). After washing with 2% FCS/PBS, secondary avidin-FITC (4 μl/sample, Becton Dickinson, San Jose, CA, USA) was added for 15 minutes at 4°C. After four times washings with PBS, the samples were analyzed by a flow cytometer (BD™LSR II) using FCS Express™Diva Software (Becton Dickinson, San Jose, CA, USA).

### Histological analyses

Dissected ankle joints were fixed in 10% paraformaldehyde/PBS, decalcified in 5% nitric acid for one week, dehydrated and embedded in paraffin. Sections (6 μm thickness, rotary microtome Accu-Cut^® ^SRM™ Sacura Finetek, Tokyo, Japan) were stained with hematoxylin and eosin (H&E), toluidine blue or safranin O/fast green. Images were captured with a coupled device camera and exported to Adobe Photoshop 7.0 (Adobe Systems, Munich, Germany). The joint damage was scored using the semi-quantitative grading and staging system [23]. The severity of damage was graded from 0 (normal joint architecture) to 6 (deformation, joint margin osteophytes formation and bone remodeling). The extent of the damage reflecting OA stage was scored from 0 (whole cartilage surface intact) to 4 (more than 50% of cartilage surface affected). The histological score of joint damage was obtained after multiplying the grade and stage scores (maximum score 24). Captured images of the joints were examined for the presence of osteophytes. The osteophyte area was measured using imaging system software (ImageJ 1.42; Research Services Branch, NIH, Bethesda, Maryland, USA) and an average value of five joint sections per group was calculated.

### Immunohistochemistry

The sections (6 μm) were permeabilized with 0.1% Triton X-100 in PBS for 20 minutes, washed with PBS and blocked with 5% bovine serum albumin/PBS for 1 h at room temperature. The endogenous peroxidase was blocked by 0.3% H_2_O_2 _in 60% methanol for 10 minutes. After washing, the sections were incubated for 40 minutes at room temperature with antibodes against RANKL (50 μg/ml; PeproTech EC), BMP2 (0.1 μg/ml), TGF-β3 (10 μg/ml), pSMAD-2 (20 μg/ml) and DKK-1 (10 μg/ml; all from Abcam, Cambridge, UK). Isotype antibodies (anti-mouse IgG or anti-rabbit IgG; Sigma-Aldrich) were used as a background staining control. After washing, the joint sections were incubated for 10 minutes with biotinylated anti-mouse or anti-rabbit IgGs (Abcam). Then streptavidin-peroxidase (1:100 diluted; Abcam) was added for 10 minutes. The sections were washed and incubated with DAB solution kit (3',3'diaminobenzididne kit, Abcam) for 10 minutes and counterstained with Gill's hematoxylin for 3 minutes. The number of cells stained positive for the examined proteins was determined by imaging system software (ImageJ 1.42; Research Services Branch, NIH, USA).

### Statistical analyses

Statistical analyses were performed using InStat3.0 and GraphicPad Prism 5.0 (GraphPad Software Inc., La Jolla, CA, USA). Data are expressed as mean ± standard deviation (SD). The histological score data and the immunohistochemistry data were analyzed using the Mann-Whitney U-test. For other data, the differences in mean values between groups were analyzed by two-tailed Student's *t-*test. Differences were considered significant when *P *< 0.05.

## Results

### Cytokine levels in the synovial extracts at Day 7 of CIOA

At Day 7 of CIOA, we found high levels of pro-inflammatory mediators MIP-1α, RANTES, soluble RANKL, TNF-α and IL-6 in the synovial extracts. The levels of anti-inflammatory cytokines IL-4 and IL-10 were nearly undetectable similarly to healthy mice (Table 1).

**Table 1:** Chemokine and cytokine levels in the joint extracts on Day 7 of CIOA

	Cytokines (pg/ml)						
	
Groups	MIP-1α	RANTES	sRANKL	TNF-α	IL-6	IL-4	IL-10
Healthy	30 ± 10	<16	<62	32 ± 20	<62	<10	28 ± 12
CIOA	720 ± 55***	320 ± 24***	300 ± 70***	760 ± 56***	540 ± 68***	28 ± 16	38 ± 10

Mice (*n *= 15 in each group) were injected i.a. with 1 U/mouse of collagenase on Day 0 and Day 2. Synovial extract was collected separately from each animal at Day 7 of CIOA and the level of several mediators was determined by ELISA; Student's *t*-test; ***P < 0.001 vs healthy mice.

CIOA, collagen-induced osteoarthritis; ELISA, enzyme linked immunosorbent assay; IL, interleukin; MIP-1α, macrophage inflammatory protein 1α; sRANKL, soluble receptor activator of nuclear factor kappa B ligand; RANTES, regulated upon activation, normal T-cell expressed, and secreted protein; TNF, tumor necrosis factor.

### Effect of glucosamine on the development of CIOA

**Figure 1:**
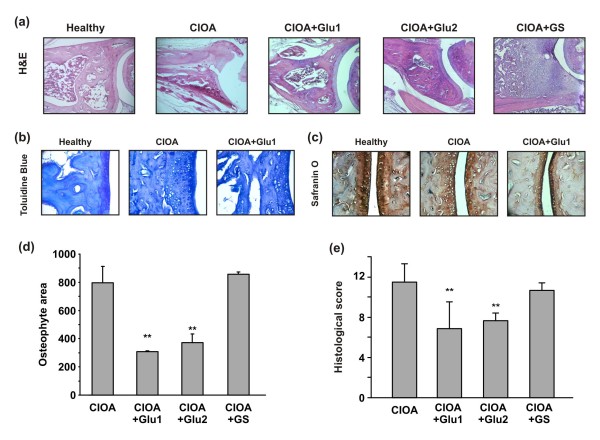
**Effect of glucosamine on the development of CIOA**. CIOA was induced by injection of 1 U/mouse collagenase at Day 0 and Day 2. After seven days, CIOA mice were orally treated with PBS (CIOA; *n *= 15), with glucosamine hydrochloride (20 mg/kg/daily; CIOA + Glu1; *n *= 15) or glucosamine sulfate (20 mg/kg/daily; GS, *n *= 10) for 20 days, or with glucosamine hydrochloride (20 mg/kg/daily) started with the second collagenase injection and lasted 20 days (20 mg/kg/daily; CIOA + Glu2; *n *= 10). A control group of non-arthritic mice were fed with PBS (healthy; *n *= 9). **(a) **Representative joint sections stained with H&E showed fissures and fractures in cartilage and bone matrix of CIOA mice at Day 30, attenuated by Glu1 and Glu2, but not by GS; magnification × 40. Staining with Toluidine blue **(b) **and Safranin O **(c) **demonstrated GAGs and PG loss in CIOA that was less pronounced in Glu1-treated CIOA mice; magnification × 100. Glu1 and Glu2 significantly reduced the osteophyte areas in CIOA mice, while GS was not effective **(d)**. Data are expressed as the mean ± SD from the evaluation of five joint sections/group; Student's *t*-test; **P <0.01 vs CIOA group. **(e) **Glu1 and Glu2 significantly decreased the total histological score in CIOA mice and nonsignificantly by GS. Total histological score was calculated using the semi-quantitative grading and staging system. Data are expressed as the mean ± SD from the evaluation of 10 joints/group; Student's *t*-test; ***P <0.001 vs CIOA group.

The severity and progression of CIOA was evaluated by scoring the histological changes in the joints (grade) and the extent of cartilage involvement in these changes (stage of CIOA). The repeated injection of 1 U/mouse of collagenase resulted in cartilage erosion and matrix loss. Histological analyses of H&E stained sections of CIOA joints showed matrix cracks and fissures extended in the deeper zone of cartilage that contributed to excavation and cartilage fractures. Reduced density of Toluidine blue staining in CIOA mice demonstrated marked loss of glycosaminoglycans (GAGs) in the cartilage (Figure 1b). Safranin O staining showed significant proteoglycan depletion in CIOA joints (Figure 1c). In several CIOA joints we were able to detect osteophyte areas indicative of bone repair processes, initiated as a result of severe cartilage and matrix loss (Figure 1d). All together our data showed the development of moderate OA, according to the total histological score of 11.4 ± 4.5 (*n *= 15, Figure 1e).

CIOA mice were treated with glucosamine hydrochloride for 20 days, starting with (Glu2) or 7 days after the second collagenase injection (Glu1). The administration of the drug under two different schedules (Glu1 and Glu2) had a beneficial effect on the development and progression of CIOA as demonstrated by inhibited joint damages (Figure 1a) and reduced total histological score (Figure 1e). Mice treated with Glu1 showed less exerted bone erosion, matrix fissures, GAG and PG loss (Figure  1b, c), and osteophyte areas (Figure 1d). The administration of GS under the schedule of Glu1 group for 20 days did not inhibit cartilage erosions, matrix loss and osteophyte formation (Figure 1a) and failed to improve histological signs of disease (Figure 1e).

In another set of experiments glucosamine hydrochloride was administered after Day 7 of injection with a higher dose of collagenase (2 U/mouse at Day 0 and at Day 2). The treatment lasted 20 days (Figure S1b in Additional file 1). At Day 30 all CIOA joints showed bone remodeling and repair with extensive osteophyte and fibrocartilage formation, denudation, deformation of articular surface and changes in the joint architecture (Figure S1a in Additional file 1). A high histological score of 22.3 ± 5.2 (maximum 24) demonstrated the development of severe OA. The administration of glucosamine decreased osteophyte and fibrocartilage areas in CIOA joints, although this effect did not reach statistical significance and was observed for some but not for all joint sections (Figure S1b in Additional file 1).

### Glucosamine altered the chemokine and interleukin secretion in the synovial extract of CIOA mice

**Figure 2:**
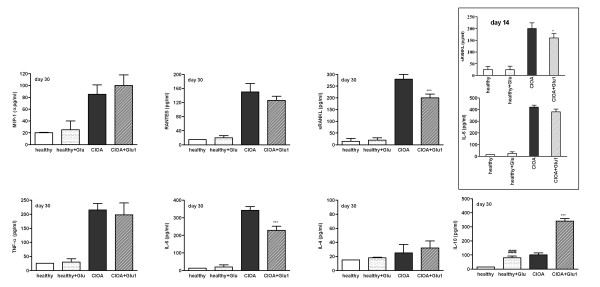
**Effect of glucosamine on the synovial level of soluble RANKL, IL-6 and IL-10**. Joint extracts were collected from individual mice treated with PBS (healthy, *n *= 9), non-arthritic mice treated with glucosamine hydrochloride for 20 days (20 mg/kg/daily; healthy + Glu, *n *= 10), CIOA mice (*n *= 15) and CIOA mice treated with glucosamine hydrochloride for 20 days, starting from Day 7 (20 mg/kg/daily; CIOA + Glu1; *n *= 15). The levels of particular cytokine (MIP-1α, RANTES, soluble RANKL, TNF-α, IL-6, IL-4 and IL-10) on Day 14 (insert) and on Day 30 of CIOA were determined by ELISA and expressed as picograms/ml supernatant. Data represent the mean ± SD of two independent experiments involving from 9 to 15 animals/group; *P < 0.05; ***P < 0.001 CIOA vs CIOA+Glu1; ^###^*P *< 0.001 vs healthy. Student's *t*-test.

Several cytokines can affect the severity and progression of OA. We evaluated the level of MIP-1α, RANTES, TNF-α, soluble RANKL, IL-10 and IL-4 in the synovium extracts on Day 30 of CIOA (Figure 2). In healthy mice the presence of all mediators was in negligible amounts similar to the group of non-arthritic mice, treated with Glu1 except for IL-10, whose level was elevated. Increased levels of MIP-1α, RANTES, TNF-α, soluble RANKL and IL-10 were established in CIOA mice. Glucosamine markedly enhanced IL-10 in the joint extracts, while MIP-1α, RANTES and TNF-α were not affected. The administration of the drug for seven days (or Day 14 of CIOA, insert in Figure 2) significantly decreased soluble RANKL. Low levels of sRANKL were also detected after long term treatment (20 days) of CIOA mice with glucosamine (Figure 2). In ongoing OA (insert Figure 2), the drug did not affect the level of IL-6, but was able to reduce it after prolonged administration (20 days; Figure 2).

### Glucosamine decreased the number of Ly6G neutrophils and CD3 T cells in the synovial extract

**Figure 3:**
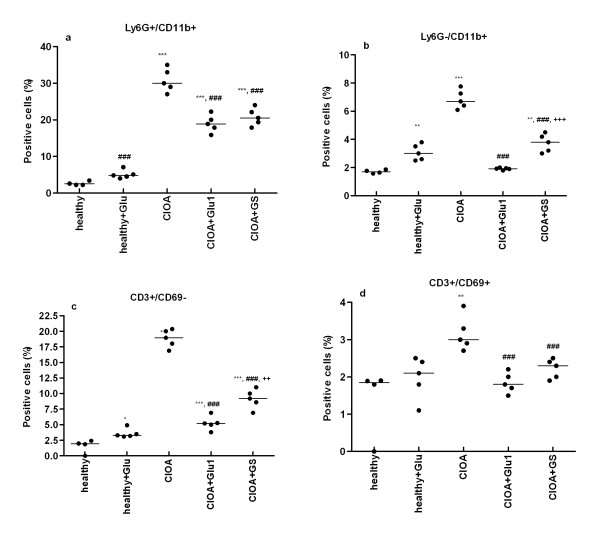
**Phenotype of cells in the synovial extract at Day 30 of CIOA**. Joint extracts were collected from mice treated with PBS (healthy, *n *= 5), nonarthritic mice treated with glucosamine hydrochloride for 20 days (20 mg/kg/daily; healthy + Glu, *n *= 5), CIOA mice (*n *= 5) and CIOA mice treated with of glucosamine hydrochloride for 20 days, starting from Day 7 (20 mg/kg/daily; CIOA + Glu1; *n *= 5) or with GS (20 mg/kg/daily; CIOA + GS, *n *= 5). Samples were obtained separately from each mouse. Individual data were presented in dot-plot graphs. The median value is shown in the graphs with line. *P < 0.05; **P < 0.01; ***P < 0.001 vs healthy; ^###^*P *< 0.001 vs CIOA group; ^++^*P *< 0.01; ^+++^*P *< 0.001 CIOA + Glu1 group vs GS. Student's *t*-test.

We performed flow cytometry analyses of synovial cells for the surface expression of CD3, CD69, Ly6G and CD11b. At Day 30 of CIOA around 30% of synovial cells expressed neutrophil marker Ly6G, significantly reduced in Glu1 and GS-treated groups (Figure 3a). Low frequencies of single CD11b cells were found in GS and Glu1-treated groups (Figure 3b). CD3 expressed on 19% of synovial CIOA cells was greatly reduced by Glu1 and more slightly by GS (Figure 3c). Similar tendency was observed in regard to double positive CD3+/CD69+ cells. The number of activated cells (3%) was decreased in the synovial extracts of Glu1- and GS-treated groups (Figure 3d).

### Glucosamine changed the expression of TNF-αR and IFN-γR on synovial CD3 T cells

**Figure 4:**
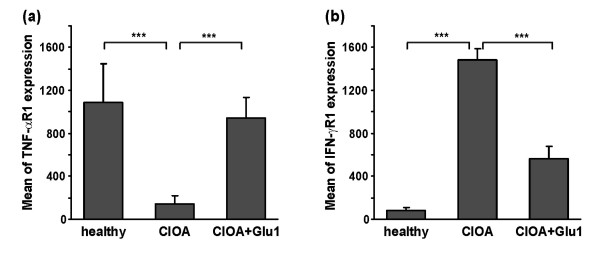
**Glucosamine up-regulated TNF-αR1 and down-regulated IFN-γR1 expression in CIOA on synovial CD3 T cells**. Synovial cells were obtained at Day 30 from joint extracts of healthy (*n *= 5) mice, CIOA mice treated with PBS (CIOA; *n *= 5) and CIOA mice treated with glucosamine (CIOA + Glu1, *n *= 5). Synovial cells were stained with PE-labeled antibody against CD3 and with biotinylated antibodies against TNF-αR1 and IFN-γR1 followed by avidin-FITC staining and were subjected to flow cytometry. Data represent the mean of fluorescence expression ± SD from two independent experiments involving five mice/group; Student's *t*-test; ***P < 0.001.

Synovial extracts from CIOA mice contained CD3 positive T cells that can respond to pro-inflammatory cytokines like TNF-α and IFN-γ after engagement of the particular receptor. Thus, we next evaluated the expression of TNF-αR and IFN-γR on CD3 T cells. In order to perform correct flow cytometry analyses, the expression of both receptors was evaluated within gated CD3 positive cell population in all groups (Figure 4). In healthy mice synovial CD3 T cells expressed TNF-αR1 (mean expression of 1,006 ± 345), while CIOA mice showed low surface expression of the receptor (mean expression of 95 ± 12). In glucosamine-treated mice with CIOA TNF-αR1 expression was comparable to that in healthy mice (mean expression of 915 ± 102) (Figure 4a). Opposite to these findings, we observed low expression of IFN-γR1 on CD3 T cells in healthy mice. The surface expression of IFN-γR1 increased in CIOA mice (mean expression of 1,496 ± 213) which was down-regulated by glucosamine (mean expression of 595 ± 87) (Figure 4b).

### Effect of glucosamine on the percentage of RANKL positive synovial and peripheral CD3 T cells

**Figure 5:**
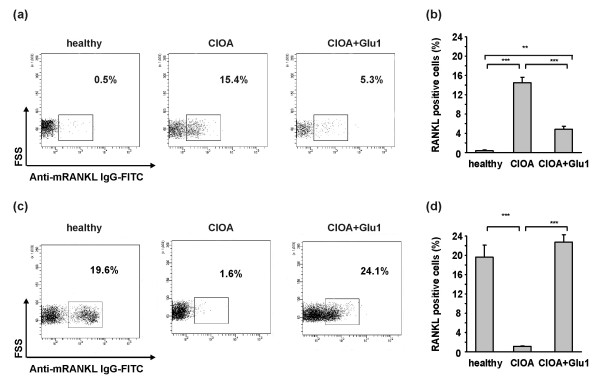
**Effect of glucosamine on the percentage of RANKL positive synovial and peripheral CD3 T cells**. Synovial cells and PBMCs were obtained at Day 30 from healthy (*n *= 5) mice, CIOA mice treated with PBS (CIOA; *n *= 5) and CIOA mice treated with glucosamine hydrochloride (CIOA + Glu1, *n *= 5). The cells were stained with PE-labeled antibody against CD3 and with biotinylated antibody against RANKL followed by avidin-FITC staining and were subjected to flow cytometry **(**a**)** Representative data showing the inhibitory effect of glucosamine on the number of RANKL positive synovial CD3 T cells, **(**b**)** Glucosamine suppressed the RANKL expression of synovial CD3 T cells. Data represent the mean of positive cells ± SD from two independent experiments involving five mice/group; Student's *t*-test; ***P < 0.001, **(**c**)** Representative data showing increased percentage of RANKL positive peripheral CD3 T cells in glucosamine-treated CIOA mice, **(**d**)** Glucosamine increased the RANKL expression of peripheral CD3 T cells. Data represent the mean of positive cells ± SD from two independent experiments involving five mice/group; Student's *t*-test; **P < 0.01, ***P < 0.001.

In the next experiments synovial CD3 T cells were subjected to flow cytometry analysis for RANKL expression. At Day 30 of CIOA, almost all CD3 positive cells expressed RANKL (15.4%; Figure 5a). Glucosamine significantly reduced the number of RANKL positive synovial CD3 T cells (Figure 5b). In the periphery CD3 T cells in all experimental groups were around 55% of total cell population (healthy mice - 50.2 ± 10.2; CIOA mice - 57.3 ± 15.4 and CIOA+Glu - 60.0 ± 6.2). In CIOA only 1.6% of peripheral CD3 T cells were RANKL positive compared to 19.6% in healthy mice and 24.1% in glucosamine-treated group (Figure 5c). Glucosamine rendered the percentage of RANKL positive CD3 T cells to the values observed in healthy mice (Figure 5d).

### Effect of glucosamine on the expression of markers for bone erosion and remodeling

**Figure 6:**
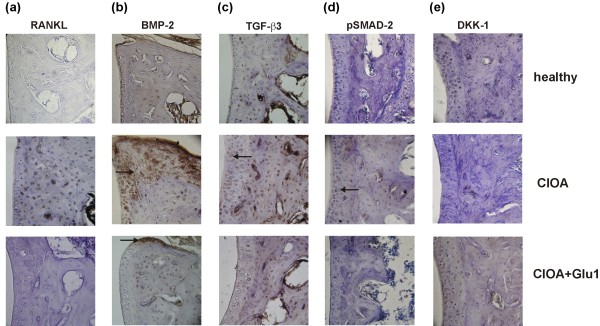
**Glucosamine altered the expression of bone resorption and bone remodeling markers in the CIOA joints**. Joints of healthy mice, CIOA mice treated with PBS (CIOA) and CIOA mice treated with glucosamine hydrochloride (CIOA + Glu1) were fixed, decalcified, dehydrated and embedded in paraffin. The sections (*n *= 10/mice) were stained with antibodies against RANKL, BMP-2, TGF-β3, pSMAD-2 and DKK-1. The specific staining was detected using a peroxidase-DAB staining kit. The positive staining for RANKL in the joints **(a)**, BMP-2 in osteophyte areas **(b) **and TGF-β3 **(c)**, pSMAD-2 **(d) **and DKK-1 **(e) **in cartilage of CIOA mice untreated or treated with glucosamine was indicated with arrows; magnification × 100.

The progression of OA is often due to the lack of balance between bone resorption and formation. We next evaluated the expression of RANKL, a molecule characteristic for bone resorption and of BMP2, TGF-α3, pSMAD-2 and DKK-1, molecules indicative for bone repair and remodeling. The expression of these markers in the joints is shown in Figure 6 and Table 2. We found single RANKL positive cells in healthy mice. High RANKL expression was determined at Day 30 of CIOA and in all joint sections with extensive bone erosion. Glucosamine reduced significantly RANKL positive cells in CIOA joints (Figure 6a, Table 2). The direct action of glucosamine on osteoclasts was determined *in vitro*. Bone marrow cells were differentiated with M-SCF and RANKL in the presence of increasing concentrations of the drug. Glucosamine inhibited in a dose-dependent manner osteoclast differentiation (Figure S2 in Additional file 2). Immunohistochemistry analysis demonstrated BMP-2 staining of osteophyte areas in CIOA joints that was exerted less in glucosamine-treated group (Figure 6b, Table 2). TGF-α3 positive cells were increased in cartilage of CIOA mice compared to healthy and glucosamine fed mice (Figure 6c). Downstream signaling of TGF-α3 involved phosphorylation of SMAD-2. The positive staining for pSMAD-2 found in cartilage of healthy mice was two-fold greater in CIOA joints. Glucosamine down-regulated the expression of pSMAD-2 (Figure 6d, Table 2). Another protein involved in bone formation is DKK-1, a specific molecule that blocks Wnt signaling. Expression of DKK-1 was observed in healthy mice. The number of DKK-1 positive cells decreased at Day 30 of CIOA indicating a loss of regulatory signal to Wnt pathway in favor of bone formation. In the group of glucosamine-treated CIOA mice we were able to detect DKK-1 positive cells not significantly different from healthy mice (Figure 6e, Table 2).

**Table 2:** Number of cells stained positive in CIOA joints on Day 30

	Bone markers				
	
Groups	RANKL	BMP2	TGF-β3	pSMAD2	DKK1
Healthy	5 ± 1	nd	109 ± 12	18 ± 3	75 ± 12
CIOA	65 ± 12	136 ± 21	145 ± 22	36 ± 6	32 ± 9
CIOA+Glu1	8 ± 1***	102 ± 10*	112 ± 14*	12 ± 3***	89 ± 13***

Joint sections were obtained and stained as described in Figure 6. The number of positive cells was evaluated by a computerized imaging system. Data represent the mean of positive cells ± SD from two independent experiments involving five mice/group (10 joint sections/mice); nd, not detectable; Mann-Whitney U-test; *P < 0.05, ***P < 0.001 vs CIOA group.

BMP, bone morphogenetic protein; CIOA, collagen-induced osteoarthritis; DKK1, Dickkopf-1; Glu, glucosamine hydrochloride; pSMAD-2, phosphorylated protein mothers against decapentaplegic homolog 2; RANKL, receptor activator of nuclear factor kappa B ligand; SD, standard deviation; TGF, transforming growth factor.

## Discussion

Glucosamine is an agent that improves functional activity and slows the progression of OA especially of the hip and knee. Most of the clinical studies with glucosamine, designed to treat OA have shown good or moderate symptomatic efficacy [20,24-26]. Contradictory results have been observed regarding the effectiveness of glucosamine on pain and disability in OA patients vs placebo patients [27,28]. Such discrepancy is due to the use of different formulations, as hydrochloride or sulfate salts are claimed to have various efficacies. Additionally, the interpretation of the results is complicated by differences in the trial duration and in the cohort studied. Chemically and structurally chloride and sulfate are identical and the nature of the included salt should not influence their biological effect. Glucosamine hydrochloride has no beneficial effect in regard to relieving OA pain and disability but the lack of histological data prevents firm conclusions about its effect on other disease symptoms [29]. Our histological data showed that glucosamine sulfate was less effective than glucosamine hydrochloride when both were administered under the same conditions in CIOA.

In the present study, the CIOA animal model has been chosen as relevant to the pathology of OA patients and the oral route as relevant to the clinical application of glucosamine. We compared two schedules for 20-day treatment with glucosamine hydrochloride, starting with the injection of collagenase or seven days thereafter (ongoing OA). In ongoing OA (Day 7) chemokines and cytokines were elevated in the synovium and histological signs of cartilage damage were already registered. On Day 30 the disease was defined as moderate OA, characterized by cartilage erosion, matrix loss, GAG and PG depletion, and formation of osteophytes. The results demonstrated that glucosamine ameliorated the degree of joint damage. Lower histological score in glucosamine-treated CIOA group was related to inhibited bone erosion, as shown by reduced GAG and PG loss, and limited areas of bone outgrowth at the edges of the joints.

However, limited numbers of investigations have demonstrated the precise mechanism of glucosamine action in OA. In this study the experiments were focused on the events in the joint without detailed assessment of the systemic action of glucosamine. In rat non-arthritic models the drug showed anti-inflammatory action by suppressing iNOS protein expression in the spleen, lung and peritoneal macrophages [10]. Yet, glucosamine might have systemic action, in that it also suppresses nuclear factor kappa B activity [30]. Particularly, this pathway is blocked in chondrocytes cultured in the presence of glucosamine, suggesting that it may suppress inflammatory signaling [7]. Cytokines and growth factors play an important role in the pathology of OA [31,32]. They are produced by the synovial cells, chondrocytes and inflammatory cells, and later, diffuse to the cartilage through the synovial fluid. Does glucosamine act on the inflammatory mediators in the synovium? On Day 30 we observed in CIOA mice high synovial levels of the pro-inflammatory cytokine TNF-α and elevated levels of chemokine RANTES (CCR5) and chemoattractant protein MIP-1α, an indication of persistent local inflammation. During ongoing OA, glucosamine was not able to reduce the production of MIP-1α, RANTES and TNF-α. After seven-day treatment we observed that sRANKL level in the synovium was lowered compared to untreated mice, but the IL-6 level was not affected. The substance, however, significantly lowered the level of soluble RANKL and IL-6, and increased the level of IL-10 in the joint on Day 30. This shows that various mediators play different roles through the course of the disease. RANKL is a ligand for RANK expressed on osteoclast precursors. RANKL-RANK interaction triggers the activation of NF-kB and AP-1 transcription factors that drives osteoclast differentiation [33]. While IL-10 directly inhibits RANKL-induced osteoclastogenesis [34], IL-6 plays a more complex and dual role in this process. IL-6 can suppress bone resorption by inhibiting the differentiation of osteoclast progenitors [35] and by down-regulating RANK expression on mature osteoclasts [36]. When IL-6 is at a high level, it binds to soluble IL-6R and can directly induce RANKL expression on osteoblasts [37] and on synovial fibroblasts that favor bone resorption [38]. In glucosamine-treated mice the reduction of IL-6 and the increase of IL-10 levels can inhibit the expression of RANKL in the joints. We found only a few RANKL-positive chondrocytes and synoviocytes in cartilage of glucosamine-treated CIOA mice. *In vitro *experiments showed that glucosamine dose-dependently inhibited osteoclast differentiation of bone marrow cells. The membrane-bound RANKL is expressed as a trimeric transmembrane protein [39], as a truncated ectodomain cleaved by TNF-α convertase and matrix metalloproteinase 14 [40,41] and as a primary secreted form, produced by activated T cells [42]. Our data showed that glucosamine inhibited the expression of RANKL in the joints and also reduced the level of sRANKL in synovial extract of CIOA mice. Soluble RANKL directly participate in bone erosion through its excess production by activated CD3 and CD4 cells in synovial fluid in RA patients [43]. Thus, we can hypothesize that diminished levels of sRANKL can be responsible for suppressed bone erosion in glucosamine-treated CIOA mice. If OA develops as a result of an imbalance between bone resorption and bone remodeling, the question arises whether glucosamine can influence another remodeling marker besides RANKL.

In established CIOA, we found intensive formation of osteophytes indicating a prevalence of bone remodeling. It is reported that cartilage damage is highly associated with the presence of osteophytes [44], which can be the source of pain and disability in OA [45]. It is not exactly known whether they are formed as a way to compensate for the instability of destructed bones or as a result of extensive local production of growth factors [46]. We observed that glucosamine inhibited the formation of osteophytes in CIOA joints. Simultaneously, we found fewer osteophyte areas positive for BMP-2 in the glucosamine-treated group. BMP-2, a member of the TGF-β family, can induce osteophyte formation [47] and is expressed at a late stage of endochondral ossification [48,49]. The reduced BMP-2 expression in glucosamine-treated CIOA mice showed that bone remodeling is initiated but does not progress. Thus, we evaluated the expression of another factor that can induce osteophyte formation, TGF-β3, which appears earlier than BMP2 in osteophytes [50]. Increased numbers of cartilage cells positive for TGF-β3 were found at Day 30 of CIOA. TGF-β3 signaling was active since phosphorylation of downstream molecule SMAD-2 was also detected in the CIOA joints. Davidson *et al*. have shown that TGF-β3 and pSMAD-2 expression in cartilage decreased with the progression of a collagenase-induced model of OA and is absent at late stages of severe OA [50]. Such different TGF-β3 and pSMAD-2 expression were observed in moderate and severe OA induced with different doses of collagenase. The injection of 1 U/mouse resulted in OA with signs of bone erosion and expression of TGF-β3 on cartilage cells. When severe OA is induced by repeated injection of 2 U/mouse (Figure S1 in Additional file 1) we were not able to detect TGF-β3 and pSMAD-2 positive cells in the joints, confirming the observations of Davidson *et al*. [50]. Down-regulated TGF-β3 and pSMAD-2 expression on cartilage cells may contribute to inhibited chondrogenesis, altered chondrocyte terminal differentiation and/or reduced hypertrophy of chondrocytes. In severe OA, the administration of glucosamine was neither able to affect TGF-β3 signaling in cartilage nor to inhibit bone remodeling and osteophyte formation. The data indicated that factors other than TGF-β3 and pSMAD-2 can become important in bone remodeling and repair at Day 30 of CIOA, probably BMP-2.

Wnt signaling is a key factor involved in bone formation. Wnt proteins enhance osteoblast differentiation and inhibit osteoclast formation [51-53]. DKK-1 is a negative regulator of Wnt signaling [54]. Heterozygous DKK-1(+/-) mice had increased number of osteoblasts and high rates of bone turnover [55]. In CIOA mice low expression of DKK-1 in the joint indicated increased activation of Wnt signaling in favor of bone formation. DKK-1 staining was more pronounced in glucosamine-treated CIOA mice suggesting an increase of bone resorption. Pro-inflammatory mediators like TNF-α can enhance DKK-1 expression [56]. However, glucosamine was not able to change the level of TNF-α in synovial fluid of CIOA mice. Probably, a complex interplay between osteoclasts and osteoblasts can explain this finding. DKK-1 can regulate osteoclastogenesis by enhancing RANKL/RANK interactions and by down-regulating osteoprotegerin secretion [53,57]. Thus, increased DKK-1 in the glucosamine-treated group can be a mechanism that prevents excessive bone formation in CIOA mice.

The perpetuation of the inflammatory response in CIOA is related to elevated numbers of cells in synovial extracts and particularly to influx of neutrophils. Hua *et al*. showed that glucosamine had a direct effect on neutrophil function. It inhibited superoxide generation and phagocytosis of neutrophils, and also can suppress formyl-Met-Leu-Phe-induced up-regulation of CD11b on these cells [11]. When coupled to FcγRs, CD11b can trigger p38 mitogen-activated protein kinase pathways, important for actin polymerization and chemotaxis of neutrophils [58]. A recent study has shown that neutrophils expressed RANKL [59] and can activate directly osteoclasts in a coculture system [60]. Glucosamine via inhibition of neutrophil chemotaxis to synovium can have an impact on osteoclastogenesis and bone destruction. CD11b is also expressed on monocytes, cells that are the source of osteoclast precursors. An inhibited number of CD11b positive cells in the synovial extracts from glucosamine-treated mice can result in reduced numbers of osteoclast precursors and in turn, in restricted osteoclastogenesis. The role of T cells in the pathology of OA should not be neglected as T cell infiltrates are frequently detected in the synovial membrane of patients with OA [61]. In CIOA mice we found an increased percentage of cells expressing CD3 (18%). However, only 3% of them were activated and expressed CD69. CD3 T cells showed low TNF-αR expression, even lesser than that on CD3 cells from healthy mice, while they were highly positive for RANKL. Recently, it has been shown that TNF-αR plays an important role in RANKL signaling, since it can compete with RANKL for the intracellular molecules TNF receptor associated factors 2, 5, and 6 [62]. When more TNF-αRs are engaged, RANKL signaling is less sensitive and expression of RANKL decreased. In support to this finding, we have observed up-regulation of TNF-αR but inhibited RANKL expression on synovial CD3 T cells from glucosamine-treated CIOA mice. RANKL-positive CD3 T cells in synovium can interact with osteoclasts, dendritic cells and/or neutrophils, all expressing RANK and thus, promoting local pro-inflammatory response and bone resorption. While glucosamine treatment reduced the number of synovial RANKL positive cells, such a reduction was not observed in the periphery. Probably, fewer RANKL-positive CD3 T cells infiltrate CIOA joints and thus, more of them were found in the periphery. Recently, it has been demonstrated that the recruitment of IFN-γR1 into the immunologic synapses of helper T (Th) cells correlates with their capacity to differentiate into Th1 effector cells [63]. In our study, glucosamine inhibited the expression of IFN-γR1 on CD3 T cells suggesting that the drug can have an impact on Th1 cell differentiation and on the perpetuation of inflammatory processes in OA. Histologically, in the OA synovium a mixed inflammatory infiltrate consisting mainly of macrophages is observed [64]. OA synovial macrophages exhibit an activated phenotype and they mediate osteophyte formation and other OA-related pathology [65]. It is an important question: Which cell type in the OA synovium is predominantly affected by glucosamine? In order to answer the question further experiments should be conducted, possibly by depletion of neutrophils, macrophages or CD3 cells.

## Conclusions

Our data show that glucosamine acted on the arthritic process in joints through inhibition of neutrophils and, at least, partially of T cells, particularly of CD3. The substance attenuates bone resorption in moderate OA via inhibition of RANKL expression in the joint, reduction of sRANKL and IL-6 levels, and increase of IL-10 amount in the synovial fluid of CIOA mice. The drug diminishes the number of RANKL positive CD3 T cells in the synovial extract and changes their RANKL expression. Reduced TGF-β3 and pSMAD-2 signaling in glucosamine-treated mice are in favor of inhibited bone remodeling and formation of BMP-2 positive osteophytes. The substance is able to limit the excessive bone formation at the late stage of disease by increased expression of DKK-1 in the CIOA joints. The data show that glucosamine ameliorates CIOA progression by regulating the degree of bone resorption and bone remodeling.

## Abbreviations

BMP: bone morphogenetic protein; CD: cluster of differentiation; CIOA: collagenase-induced osteoarthritis; DKK1: Dickkopf-1; ELISA: enzyme linked immunosorbent assay; GAGs: glycosaminoglycans; Glu: glucosamine hydrochloride; GS: glucosamine sulfate; IFN: interferon; IL: interleukin; iNOS: inducible nitric oxide synthase; i.a.: intra-articular; M-CSF: macrophage colony-stimulating factor; MIP-1α: macrophage inflammatory protein 1α; OA: osteoarthritis; PBMCs: peripheral blood mononuclear cells; PG: proteoglycan (cartilage aggrecan); RA: rheumatoid arthritis; RANKL: receptor activator of nuclear factor kappa B ligand; RANTES: regulated upon activation, normal T-cell expressed, and secreted protein; pSMAD-2: phosphorylated protein mothers against decapentaplegic homolog 2; SD: standard deviation; TGF: transforming growth factor; TNF: tumor necrosis factor; Th: T helper cells; TRAP: tartrate-resistant acid phosphatase.

## Competing interests

The authors declare that they have no competing interests.

## Authors' contributions

PD designed and supervised the experiments, performed the statistical analysis and prepared the manuscript. NI helped design the experiments, analyzed data and reviewed the manuscript.

## Supplementary Material

Additional file 1 **Figure S1. Administration of glucosamine in severe CIOA**. ICR mice were injected with high dose of collagenase (two i.a. injections with 2 U/mouse at Day 0 and Day 2). After 7 days glucosamine hydrochloride was administered orally at a dose of 20 mg/kg for 20 days. **(a) **Representative joint sections stained with H&E showed a mild effect of glucosamine on osteophyte formation and bone remodeling at Day 30 of severe CIOA. **(b) **Histological score of CIOA joints was not significantly affected by glucosamine. The data are expressed as the mean ± SD from two independent experiments involving five mice per group. Click here for file

Additional file 2 **Figure S2. Effect of glucosamine on osteoclast differentiation *in vitro***. Bone marrow cells from healthy mice were isolated, resuspended at 1 × 10^6^/ml in MEM medium (Lonza, Verviers, Belgium) containing 10% FCS and 50 ng/ml of macrophage colony-stimulating factor and cultured for one day. Osteoclasts were generated after six days of culture with 100 ng/ml RANKL and 50 ng/ml macrophage colony-stimulating factor, in the absence or the presence of increasing concentrations of glucosamine (starting from 5 till 100 μg/ml). Tartrate-resistant acid phosphatase (TRAP) on osteoclasts was determined by TRAP staining kit (Sigma-Diagnostics, Charleston, WV, USA). The number of TRAP-positive cells was counted and the data are expressed as the mean ± SD from three independent experiments. Click here for file
