# Supplementary figures and images for: Bone resorption and remodeling in murine collagenase-induced osteoarthritis after administration of glucosamine
Source: Arthritis Res Ther. 2011 Mar 16;13(2):R44. doi: 10.1186/ar3283 (PMC3132029; doi:10.1186/ar3283)

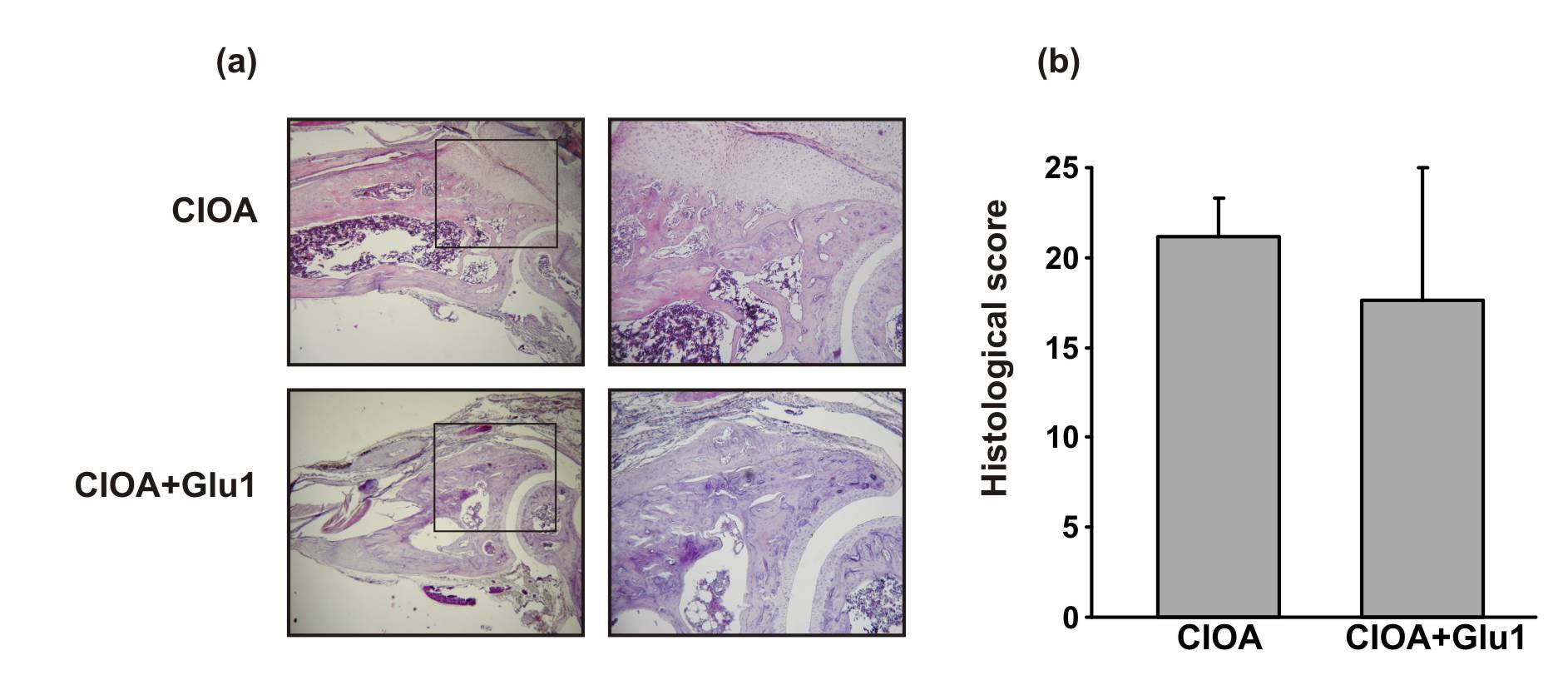

Supplement: Additional file 1 — Figure S1. Administration of glucosamine in severe CIOA. ICR mice were injected with high dose of collagenase (two i.a. injections with 2 U/mouse at Day 0 and Day 2). After 7 days glucosamine hydrochloride was administered orally at a dose of 20 mg/kg for 20 days. (a) Representative joint sections stained with H&E showed a mild effect of glucosamine on osteophyte formation and bone remodeling at Day 30 of severe CIOA. (b) Histological score of CIOA joints was not significantly affected by glucosamine. The data are expressed as the mean ± SD from two independent experiments involving five mice per group. [file ar3283-S1.JPEG]

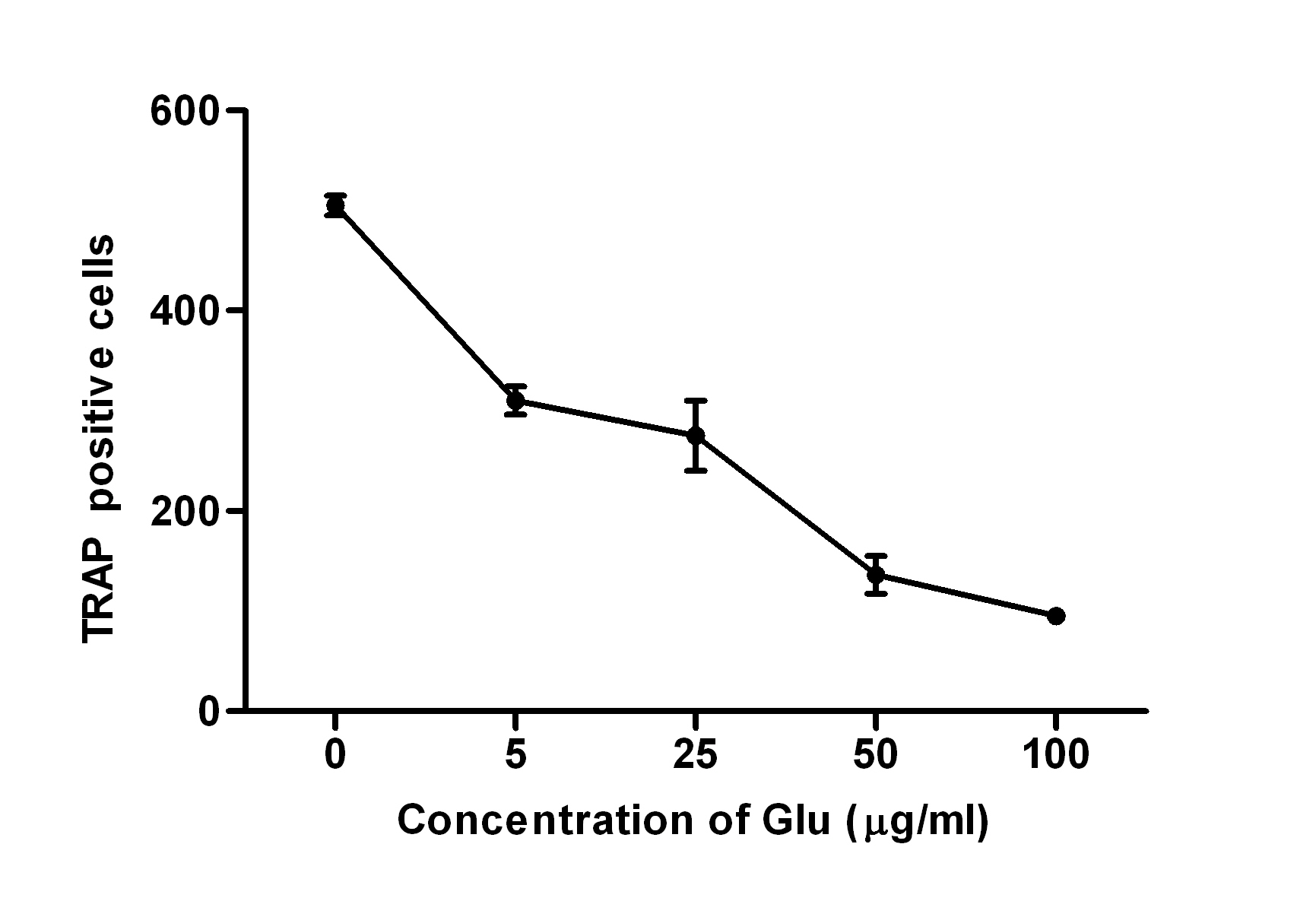

Supplement: Additional file 2 — Figure S2. Effect of glucosamine on osteoclast differentiation in vitro. Bone marrow cells from healthy mice were isolated, resuspended at 1 × 10^6^/ml in MEM medium (Lonza, Verviers, Belgium) containing 10% FCS and 50 ng/ml of macrophage colony-stimulating factor and cultured for one day. Osteoclasts were generated after six days of culture with 100 ng/ml RANKL and 50 ng/ml macrophage colony-stimulating factor, in the absence or the presence of increasing concentrations of glucosamine (starting from 5 till 100 μg/ml). Tartrate-resistant acid phosphatase (TRAP) on osteoclasts was determined by TRAP staining kit (Sigma-Diagnostics, Charleston, WV, USA). The number of TRAP-positive cells was counted and the data are expressed as the mean ± SD from three independent experiments. [file ar3283-S2.JPEG]
